# Comparison of the influences of different ventilation corridor forms on the thermal environment in Wuhan City in summer

**DOI:** 10.1038/s41598-023-40211-8

**Published:** 2023-08-17

**Authors:** Xuesong Li, Kai Lin, Yulong Shu, Xindong Lin

**Affiliations:** School of Civil Engineering, Architecture and Environment, Hubei University of Technology, Wuhan, China

**Keywords:** Climate sciences, Environmental sciences

## Abstract

The ventilation corridor is an essential element in urban planning and design to improve the climate and environment. In this paper, four forms of two ventilation corridors were set up in the southeast of Wuhan City based on its urban planning outline to quantitatively study the influences of different ventilation corridor forms on the urban thermal environment in summer. The urban micro-meteorological environment was simulated using the next-generation mesoscale Weather Research and Forecast (WRF) model coupled with an urban canopy model (UCM). Critical time-dependent meteorological values were extracted and plotted, including temperature and wind difference fields, average temperature at 2-m height, average wind speed at 10-m height, and surface energy flux. By conducting a comparative analysis of the quantitative results, the ventilation corridors in construction land are arranged at intervals in the upwind position of the city in summer, which can slightly adjust the thermal environment of the central area of the city, and have an effective regulation on the temperature of the corridors and surrounding areas during the daytime. Especially at 15:00 pm when the temperature is at its highest during the day, the temperature can be reduced by 0.8 °C. Compared to other corridors, the wind speed in and around the corridor is the strongest 11 h a day. Due to the internal arrangement of construction land, this corridor form is more advantageous in the urban land utilization. After considering comprehensively, the ventilation corridor form with construction land arranged at intervals is the preferred corridor form in the southeast of Wuhan. The experimental results can provide quantitative reference for the layout of ventilation corridors in hot inland cities located in central China.

## Introduction

Chinese cities have entered an era of rapid development in the context of rapid global urbanization. Since its reform and opening up from 1978 to 2015, China's Urbanization rate (proportion of permanent urban residents to the total population) has increased from 17.9 to 56.1%, yielding a 1.03% average annual rate of increase^1,2^. Urban construction land area has increased from 6720 km^2^ in 1981 to 49,900 km^2^ in 2014, with a mean annual rate of increase rate of 6.27%^3^. Urbanization leads to increases in the intensity of human interference. Consistent with the rise in the energy consumption of human activities and waste heat emission, there is an obvious enhancement of the urban heat island effect and an obvious reduction in air quality. To effectively relieve the urban heat island effect and improve air quality, people can establish urban ventilation corridors to introduce fresh suburban air into downtown areas while releasing urban waste gases, lowering urban internal temperatures, and purifying city air.

Urban ventilation corridors are also called urban ventilation pathways or urban air channels. After analyzing the climate characteristics, natural landscape elements, and land functional layout of the city, Hong^4^ believed that ecological green belt, river and lake system belt, and low-density construction development belt in the city should be established as a large-scale urban open space, namely urban ventilation corridor, with functions such as fresh air supply and heat discharge. It was in Germany that the world's first theoretical design and planning of ventilation corridors took place. Kress^5^ developed a German standard according to local circulation laws of urban climatology and proposed an urban ventilation system comprising affected space (GER: Wirkungsraum), compensation space (GER: Ausgleichsraum), and an air guide corridor (GER: Luftleitbahn), with defined the function and planning measures of each space. Subsequently, the academic community began to experiment with planning ventilation corridors in densely populated cities. Research topics include simulation analysis of urban ventilation capacity^6^, corridor design based on urban form^7^, and improving the ventilation capacity of urban streets and blocks^8,9^.However, there are still shortcomings in the comparative study of ventilation performance between different corridor design modes, especially at the macro scale. In terms of research methods, the academic community initially focused mainly on the site observation method^10^ and the wind tunnel test method for small area buildings^11,12^.However, the method of station observation is limited by the scarcity and uneven distribution of points. It is not universal. Due to the high simulation costs, the wind tunnel test method is not suitable for large-scale research.

Later some scholars combined measurement and numerical simulation to study the ventilation corridor's ability to introduce sea breeze and river breeze into cities, in order to improve the urban thermal environment^13,14^. Li^15^, Hong^4^, and Ren^16^ used Computational Fluid Dynamics (CFD) to simulate the effect of ventilation corridors in street canyons, and proposed that the ventilation effect can be improved by increasing street width, changing the shape of buildings along the street, and adding greenery on both sides of the street. According to the variation law of ventilation potential with the angle (between prevailing wind and street), Yin^17^ evaluated the ventilation potential of the street by using the Geographic Information System (GIS) and CFD.

Computational fluid dynamics (CFD) is only suitable for residential-scale environmental simulation due to the high computational cost. The mesoscale WRF model coupled with the urban canopy model (UCM) is more appropriate for simulating the climate environment at urban and regional scales. The WRF mode has a number of advantages, such as high simulation accuracy, strong scalability, high efficiency, good portability, and convenient use^18^. After comparing different physical schemes, different urban canopy schemes and different methods for estimating UHI (urban heat island intensity), Vogel Julian^19^ believed that the calculation results of WRF model coupled with the single-layer urban canopy model can be well consistent with the measurements with a root mean squared error of 0.86 K and a mean bias error of 0.20 K. Perini de Souza Noele Bissoli^20^ assessed the onshore and offshore wind speed fields in Bahia, northeastern Brazil, for 5 years (2015–2020) using the WRF model and detailed data of the ground station, and made wind field maps with ideal results. This paper focuses on the thermal environmental impact of ventilation corridors at the urban scale. Therefore, the mesoscale WRF model coupled with the UCM model was chosen as the research method. This paper is based on the WRF–UCM research method to quantitatively study the impact of different ventilation corridor modes in southeastern Wuhan on the urban thermal environment at the overall urban scale (mesoscale). Then, the thermal environment adjustment ability of different modes of ventilation corridor and the ventilation corridor mode suitable for Wuhan will be obtained. The research results of this paper can improve the lack of academic research on the comparison of thermal environment regulation ability of different corridor planning modes. It can also provide reference for scholars to continue the related research of ventilation corridor.

## Overview of Wuhan City

Wuhan is located in the middle and lower reaches of the Yangtze River, the transition zone between the Jianghan Plain and the low hills of the Dabie Mountains, a megacity in Central China, and an important industrial and commercial area. The middle of Wuhan is marked by the intersection of the Yangtze and Han Rivers, dividing the city into three districts: Wuchang, Hankou, and Hanyang. Wuhan has a subtropical humid monsoon climate with sufficient rainfall, sunshine, and four distinctive seasons, rain and heat in the same season. It is crowned as "the city of hundreds of lakes" as it contains 166 lakes of different sizes. In Wuhan, it is extremely hot and humid in summer, especially in July, the average temperature reaches 30℃, and a temperature above 40℃ occasionally occurs. The prevailing winds are southeasterly in summer, the wind force is mainly light air or breeze. Geographical and climatic conditions require the construction of a suitable urban ventilation environment. The basic geographic and meteorological pieces of information about Wuhan are shown in Table 1^21–23^.

**Table 1:** Wuhan City statistics.

Urban statistics project	Wuhan City statistics	
Geographical location	East longitude 113°41′–115°05′, northern latitude 29°58′–31°22′	
Overall water area	2217.6 km^2^ (2010)	
Lake area	803.17 km^2^ (2010)	
Agricultural acreage	2102.3 km^2^ (2000)	1994.4 km^2^ (2013)
Population	7,491,900 (2000)	10,220,000 (2013)
Total area of Wuhan	8467.11 km^2^ (2000)	8494.41 km^2^ (2013)
Urban built-up area	209.99 km^2^ (2000)	534.28 km^2^ (2013)
Annual extreme minimum temperature	−4.7 °C (2000)	−7.2 °C (2013)
Annually extremely maximum temperature	39.3 °C (2000)	39.5 °C (2013)
Mean temperature in summer (Jun. Jul. Aug.)	29 °C = (27.2 + 31.1 + 28.7)/3	29.1 °C = (2.16 + 30.6 + 30.6)/3

**Figure 1:**
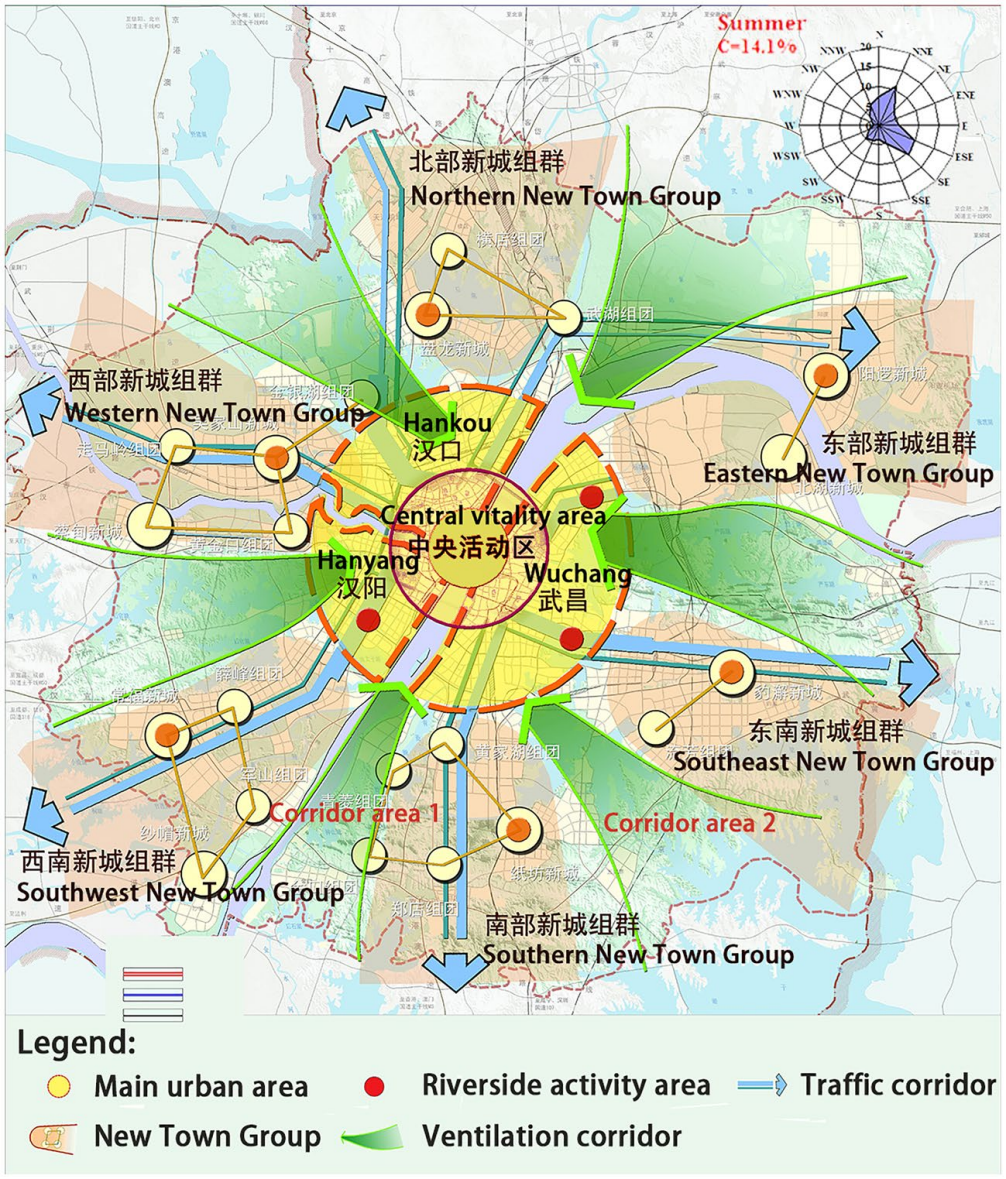
Six green wedges in Wuhan development plan. The image was created by the research team using Arcgis 10.5 (https://www.esri.com/en-us/home) based on the "Overall Land and Space Planning of Wuhan City (2021–2035)". This document was publicly available from July 14, 2021 to August 13, 2021 on the website of the People's Government of Hubei Province, China. (https://www.hubei.gov.cn/hbfb/szsm/202107/t20210715_3646185.shtml).

As a major transportation hub and mega-city in China, the rapid development of construction in recent years has caused severe industrial pollution and exacerbated the urban heat island effect. In order to improve the urban climate, the Wuhan Municipal Bureau of Natural Resources and Planning has formulated the Wuhan Urban Master Plan for 2009–2020, which clearly states that the development of the city must ensure the spatial structure of the six green wedges (ventilation corridors) (Fig. 1). In 2013, Wuhan University, Wuhan Land Resources and Planning Information Center, and the Chinese University of Hong Kong and other institutions jointly conducted a study on planning and management of Wuhan urban ventilation corridor^24^. At present, the research and construction of ventilation corridors in Wuhan mainly focus on the spatial layout of the corridor system. The specific construction mode of the ventilation corridor can be improved. It is necessary to find a more suitable corridor construction mode for Wuhan to improve the thermal environment regulation effect of the existing urban corridor system. In 2019, when our team used the WRF model to study the impact of urban construction land expansion on the thermal environment^25^, it was found that in summer, along the direction of the prevailing winds (come from the southeast of the city), there would be obvious low temperature zones in the potential corridors and surrounding areas during special periods. The southeast of Wuhan is an important area of science, education, culture, and a industry in central China. The high-tech development zone is located here. The expansion of urban construction land to the southeast has begun. Therefore, it is necessary to study the thermal environment regulating ability of different ventilation corridor modes with the windward (southeast) part of Wuhan in summer as the simulation area. It can provide a theoretical basis for improving the regulating effect of the existing ventilation corridor system in Wuhan.

## Research method and cases setting

### WRF + UCM digital model

The Weather Research and Forecasting Model (WRF) is a unified mesoscale weather prediction model jointly developed by the U.S. National Center for Environmental Prediction (NCEP), the National Center for Atmospheric Research (NCAR), and other research facilities. It has a variety of physical parameterization schemes, including microphysical process, cumulus convection, boundary layer, radiation process, land surface process, near-surface parameterization scheme, etc. These schemes are used to reflect the physical process of the factors in the study area, and can meet the needs of most mesoscale weather research, such as wind thermal simulation, air quality simulation, air-sea coupling, etc. Since version 2.2, Urban Canopy Model (UCM) has been coupled into WRF model to improve the rough description of lower boundary conditions in WRF and provide more accurate simulation results for urban areas with complex morphology^26^. The UCM model exists as a module in WRF. When running in WRF mode, it can be successfully called by modifying the options of namelist file, importing the UCM land classification map, and modifying the UCM urban land parameters in three steps. The UCM model considers the geometric characteristics of a city, the shielding effect of buildings against radiation, and the reflection of buildings on short-wave and long-wave radiation. The effect of different urban surfaces on the atmosphere can be reflected by adjusting urban surface parameters such as land use type, reflectivity, roughness, etc.Based on the WRF–UCM model, this paper can successfully change the underlying surface properties of the study area to explore the changes of meteorological elements when using different ventilation corridor modes. Based on this, the ability of the different ventilation corridor modes to regulate the thermal environment can be further quantified.The physical schemes of the research model are shown in Table 2.

**Table 2:** Physical schemes in the WRF + UCM coupled model.

Physical process	Physical scheme
Micro physical	New Thompson scheme
Long-wave radiation	RRTM scheme
Short-wave radiation	Goddard scheme
The surface layer	MYJ Monin–Obukhov scheme
Land surface	Monin–Obukhov(Janjic Eta) scheme
cumulus physical	Kain-Fritsch (New Eta) scheme

The UCM model and parameterization scheme used in this paper are all part of the WRF model. The UCM model is a specific module in the WRF. The model constructs the mechanism for simulating the meteorological elements of the urban canopy (atmosphere below the height of buildings). Using any module in WRF mode (such as the UCM module, the atmospheric simulation module, the hydrological simulation module) requires not only calling that module, but also selecting the appropriate physical scheme. These schemes are used to reflect the physical process between simulated elements. These schemas are not included with each module in WRF mode. Instead, they are juxtaposed. Taking the UCM model as an example, the physical schemes selected in this paper are used to reflect the changes of physical processes between urban building surface, atmosphere, urban blue-green space and other elements in the urban canopy area.

### Description of simulation range

**Figure 2:**
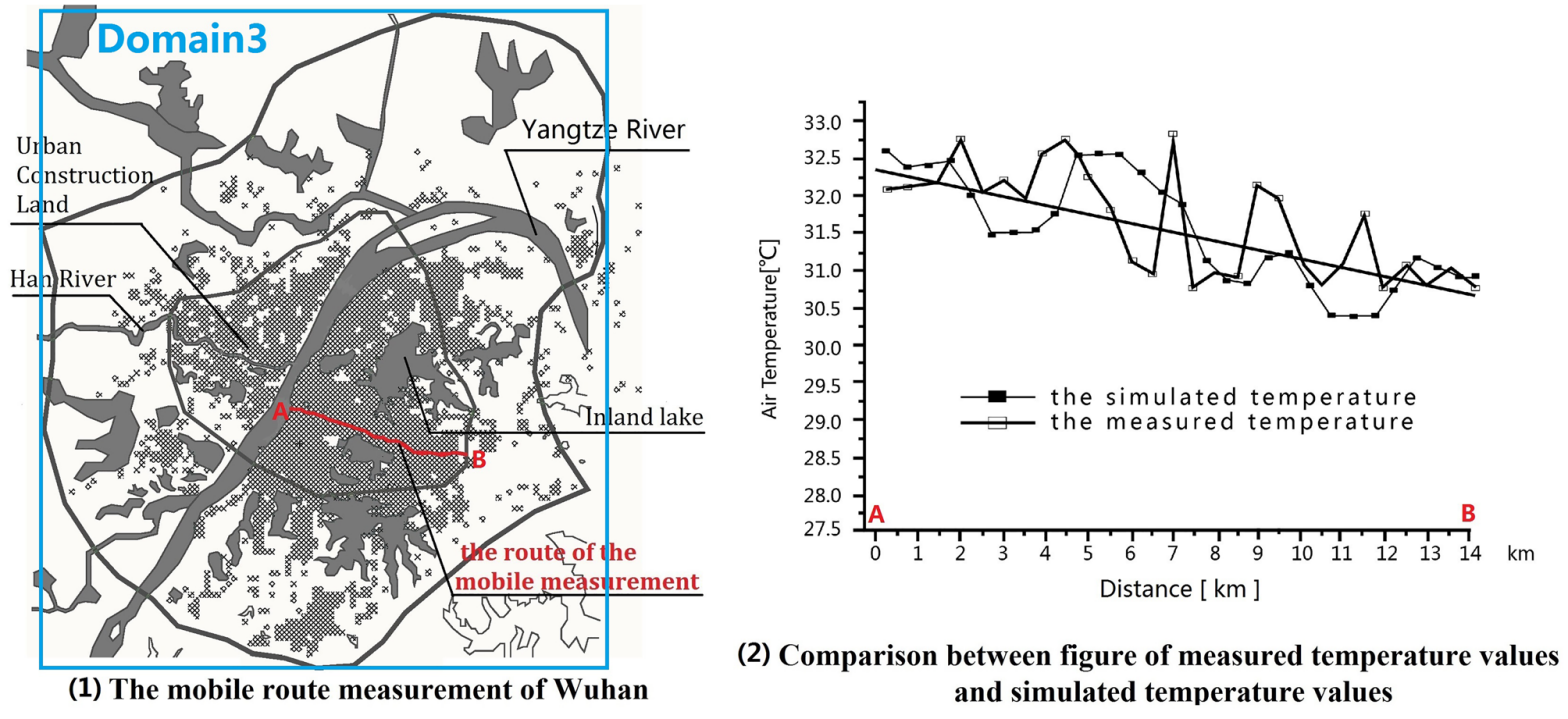
A comparative diagram between measured 2-m temperature values and simulated 2-m temperature values along the mobile route in Wuhan. Image (1) was generated by the research team using Arcgis 10.5 (https://www.esri.com/en-us/home) based on Google Remote Sensing images (http://www.gditu.net/). Image (2) was generated by the research team using Origin 2021(https://www.originlab.com).

We included all areas within the Wuhan outer circle in Domain 3, with the central point set at 113°30' E, 30°50' N. The horizontal grid resolutions of Domain 3, Domain 2, and Domain 1, are 0.5 km, 1.5 km, and 4.5 km, respectively. Domain 1 provides boundary conditions for Domain 2, and Domain 2 provides boundary conditions for Domain 3. The model's vertical domain extends from the ground to 20 km and is divided into an average of 35 layers. The grid of the computational domain is shown in Table 3, and the computational area and location are shown in Figs. 2(1) and 3(1). This study focuses on urban meteorology from the ground to a height of 30 m and within Domain 3. The original Wuhan land-use data are collected from the land-use and land-cover dataset of the United States Geological Survey (USGS) and revised according to the current situation. In USGS-24 global land use information data, Wuhan land-use data covers eight categories, namely urban, dryland crop (Dryland C. P.), irrigation crop (Irrg. C. P.), crop/grass mosaic (C/G. Mosaic), crop/wood mosaic (C/W. Mosaic), grassland, shrub land, and water (Table 4).

**Table 3:** Computational domains and grid detail.

	Domain size (X*Y*Z)	Grid number	Grid size
Domain 1	459*540*20	102*120*35	4.5 km
Domain 2	153*180*20	102*120*35	1.5 km
Domain 3	51*60*20	102*120*35	0.5 km

**Figure 3:**
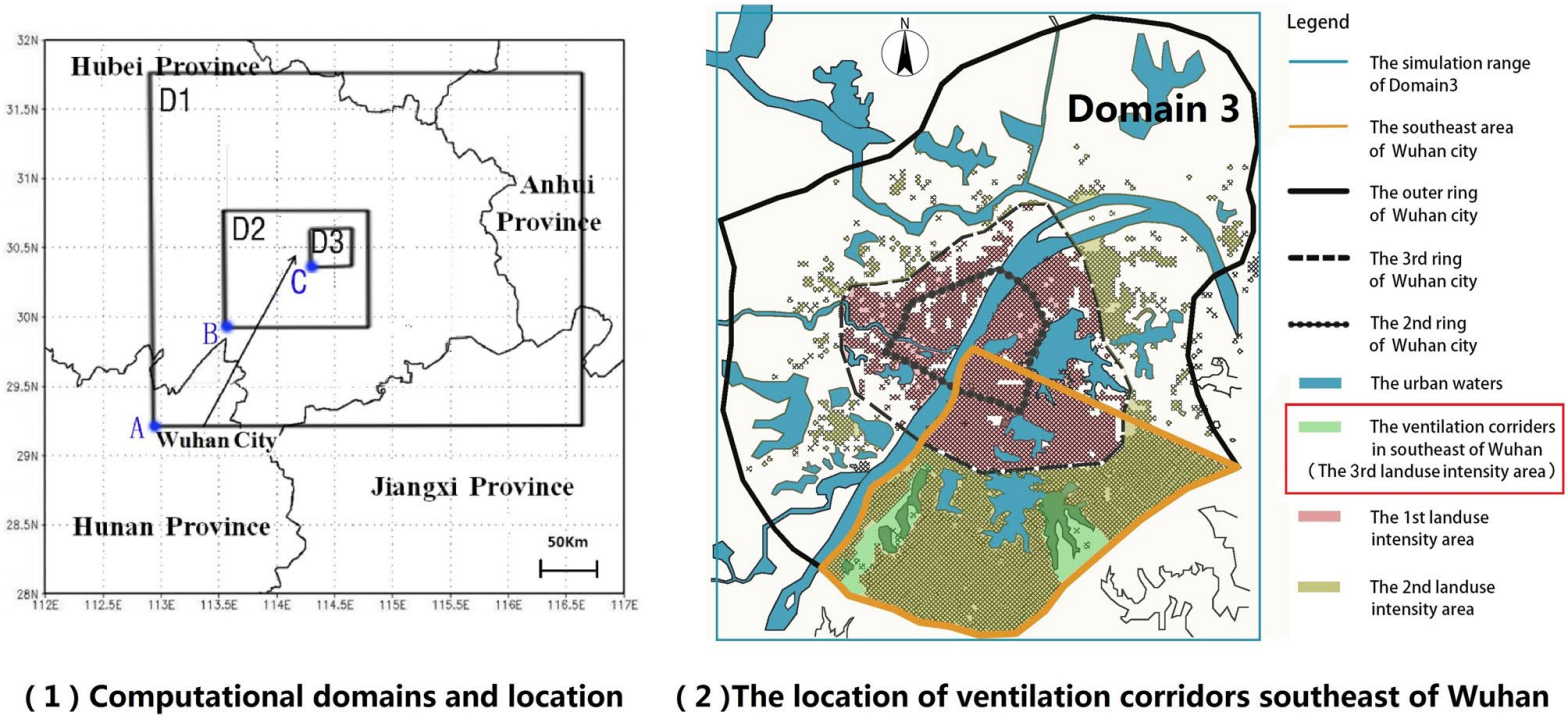
Three computational domains and two ventilation corridors in the southeast of Wuhan. Image (1) and (2) were generated by the research team using Arcgis 10.5 (https://www.esri.com/en-us/home) based on Google remote sensing images. (http://www.gditu.net/).

**Table 4:** Surface parameters of USGS land-use and land-cover data.

Land use	Albedo (%)	Moisture avail. (%)	Emissivity (% at 9 mm)	Roughness length (cm)	Thermal inertia (cal cm^−2^ K^−1^ s^−1/2^)
Urban	15	10	88	80	0.03
Dryland C.P	17	30	98.5	15	0.04
Irrg. C.P	18	50	98.5	15	0.04
C/G.Mosaic	18	25	99	14	0.04
C/W.Mosaic	16	35	98.5	20	0.04
Grassland	19	15	98.5	12	0.03
Shrubland	22	10	88	10	0.03
Water bodies	8	100	98	0.01	0.06

*C/G.Mosaic* cropland/grassland mosaic, *C/W.Mosaic* cropland/woodland mosaic.

### Model verification

The WRF model coupled with the simple single-layer urban canopy model has been applied in urban meteorology and urban planning research. Many scholars have compared the simulated values with the measured values, and the results show a good agreement. This model can effectively deduce the change characteristics of various meteorological elements, and meet the precise requirements of urban scale climate research^27–29^^.^ In order to confirm the feasibility of the WRF + UCM model, our research group conducted Wuhan urban meteorological simulation, and compared it to the mobile measured value (the parameter settings are shown in Table 5). Because the movement of vehicles on the measured route affected the wind speed and direction, we only select the temperature value for comparison. The mobile measured time was August 15, 2011, and the route was nearly 20-km long in the southeast of Wuhan City (see Fig. 2(1)). Comparing the corrected measured temperature values with the simulated temperature values at a height of 2-m: the maximum deviation is 1.173 ℃, the minimum deviation is 0.237 ℃, and the average deviation is 0.673 ℃. The results showed that the wind environment was more sensitive to on-site conditions, and the measured and simulated temperature values were in good agreement with each other, and their variation trend were consistent (see Fig. 2 (2)). Therefore, the simulation of WRF + UCM is feasible for this study. Mobile measurement, data acquisition and modification are detailed in "Observing Summer Urban Heat Island from City Center to Edge—A Case Study of Wuhan"^30^.

**Table 5:** Intensities of land use and parameter settings.

	The 1st intensity of land use	The 2nd intensity of land use	The 3rd intensity of land use			
	Case 1/2/3/4	Case 1/2/3/4	Case 1	Case 2	Case 3	Case 4
Roof_level (m)	30	21	21	0	10	21
Greening rate (%)	30	30	30	100	60	30
Frc_urb (fraction)	0.70	0.70	0.70	0	0.40	0.70
Roof_width (m)	55.0	30.0	30.0	0	20.0	30.0
Road_width (m)	20.0	20.0	20.0	20.0	20.0	20.0
Anthropogenic heat (W/m^2^)	90	50	50	0	30	50

### Cases setting

**Figure 4:**
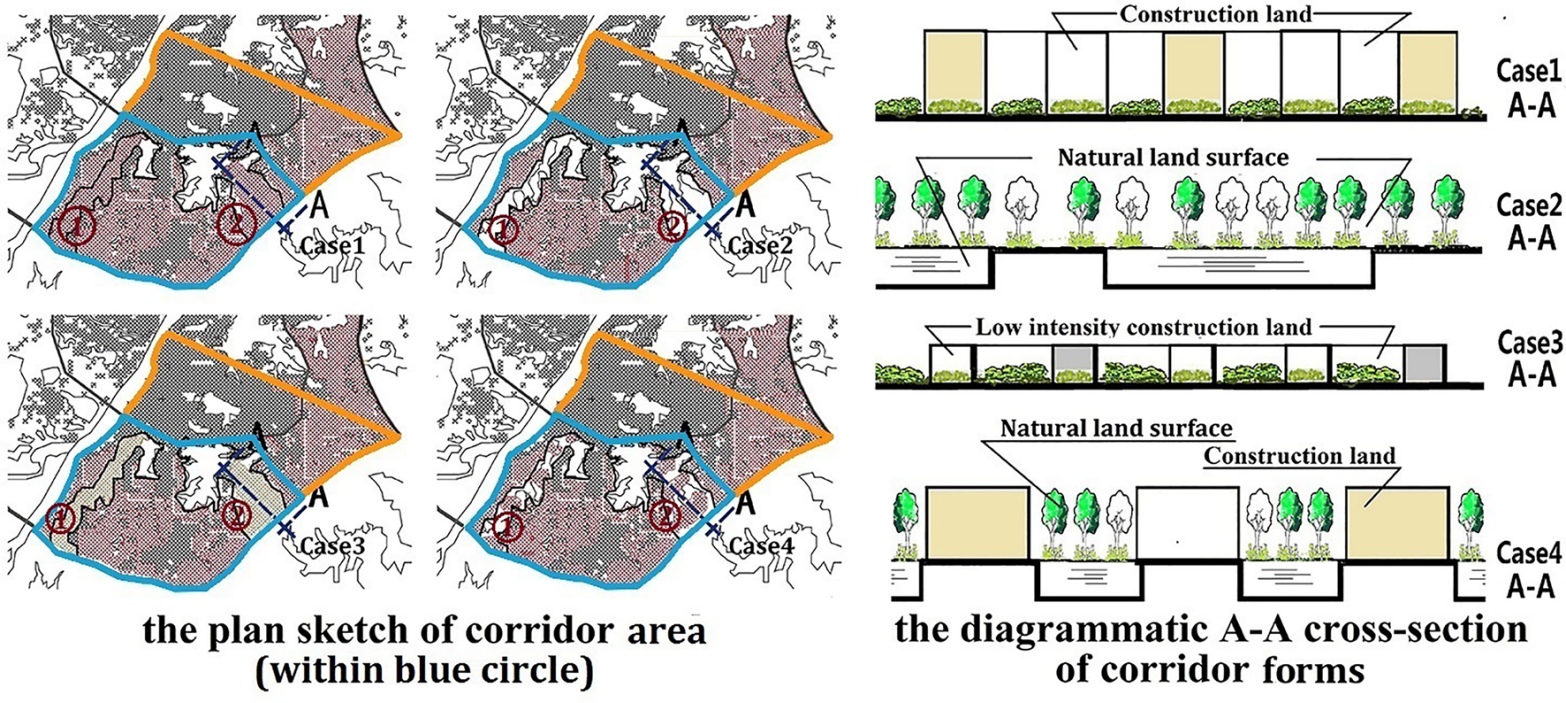
The plan sketch and the diagrammatic A–A cross-section of corridor forms.The image was generated by the research team using Arcgis 10.5 (https://www.esri.com/en-us/home) and Adobe illustrator 2021. (https://www.adobe.com).

This paper focuses on the impact of ventilation corridors on the urban climate in summer. Due to the prevailing southeast wind during the summer months in Wuhan, this study will focus solely on the issue of ventilation corridors in the southeastern region of the city. The original landsurface in the corridor in the southeast of the city is dominated by water. Figure 3(2) shows the location of ventilation corridors in Southeast Wuhan. Figure 4 shows the plan sketch and a diagrammatic cross-section of the four corridor forms. The following section provides detailed information on each case setting.

Case 1—no-green corridor form: the same construction land is set in the ventilation corridors as in the surrounding area, which is essentially a case without adapted ventilation corridors.

Case 2—pure-green corridor form: the original water body and vegetation are kept in the ventilation corridors without changing the nature of the original land.

Case 3—low-density construction land corridor form: lower-intensity construction land is set in the ventilation corridor, and the greening rate is higher.

Case 4—interval construction land corridor form: construction land with the same intensity as the surrounding land is set in the ventilation corridor at intervals, and the original water body and vegetation are reserved at intervals.

Due to WRF software's restriction on parameter settings of urban land use, the city can be divided into three zones with different intensities of construction land (see Fig. 3(2)). The built-up area within the third ring is the zone with the first intensity of construction land (the1st landuse intensity area: fixed value); the area between the third ring and outer ring is the zone with the second intensity of construction land (the 2nd landuse intensity area: fixed value); the construction land within the ventilation corridor is set as the zone with the third intensity of construction land (the 3rd landuse intensity area: variable values). The parameter values of the three intensities of construction land are shown in Table 5. Each construction land intensity index shall be set after average weighting according to the urban land intensity index by the *Interim Provisions on the Administration of Construction Land Intensity in Wuhan's Main Urban Areas*.

## Research results and discussion

### Research results

In order to reduce the calculation load, after screening the summer meteorological data of Wuhan from 2005 to 2015 (http://www.cma.gov.cn/), we determined a week (2011.08.11 to 2011.08.17) that can reflect the summer climate characteristics of Wuhan (high temperature, less rain, and weak wind) for simulation. Among the simulation results, some meteorological values and surface energy values are selected for display and analysis. The meteorological values in this paper correspond to Beijing time, hereafter indicated by local time (LT); for example, midnight is given by either 0000 or 2400 LT and noon by 1200 LT.

### A. Analysis of temperature and wind difference fields in the urban area

The Earth's surface absorbs the sun's radiation during the day and releases heat into the atmosphere after sunset. The variable nature of Earth's surface causes it to absorb and release heat differently. In order to intuitively reflect the urban climate difference caused by various ventilation corridors, the temperature-wind fields of the three cases (Case 2\Case 3\Case 4) with ventilation corridors are respectively subtracted from the temperature-wind field of the case without ventilation corridor (Case 1), and the temperature-wind difference fields are obtained as follows: (Case 2–Case 1, Case 3–Case 1, Case 4–Case 1) to reflect the climatic effects of ventilation corridors.In the temperature-wind difference field, the temperature value is at the height of 2 m, and the wind value is at the height of 10 m. The temperature difference fields at five times (0400, 1000, 1600, 2000, and 2300 LT) on August 15, 2011 are selected to represent the temperature difference fields in the wee hours, forenoon, afternoon, evening, and midnight.

**Figure 5:**
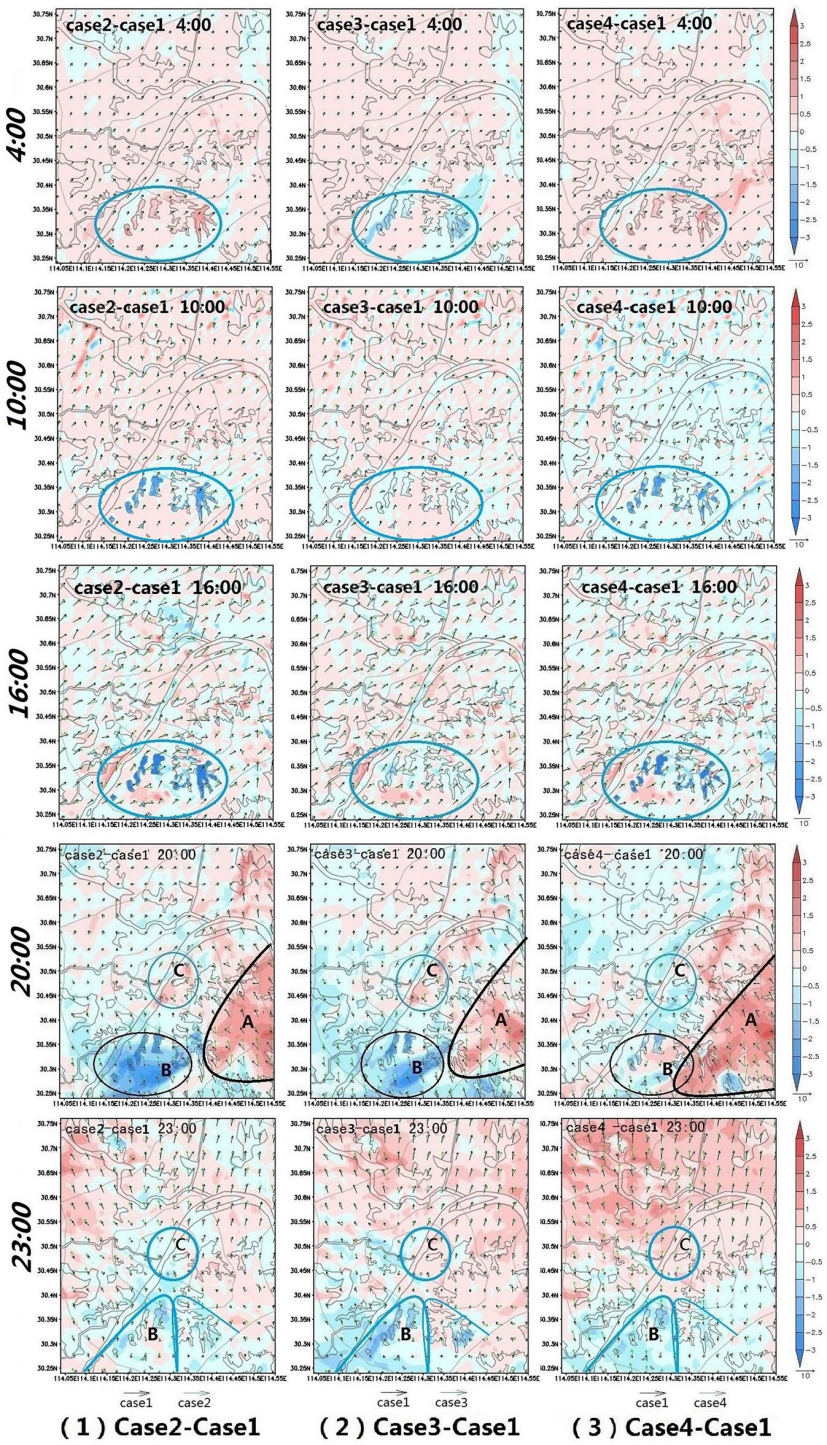
Temperature-wind difference field at multiple moments. The image was generated by the research team using GrADS 2.2.0. (http://cola.gmu.edu/grads/). The data was extracted from the simulation results of the WRF-UCM model.

From midnight to sunrise (0000–0600 LT), surface heat release gradually stabilizes. In and around the corridor, there are positive temperature difference patches in the temperature difference fields (Case 2–Case 1 and Case 4–Case 1), and slightly negative temperature difference patches in the temperature difference field (Case 3–Case 1) (Fig. 5; 0400). It can be seen that compared with the construction land (Case 1), when the patches in the corridor are water (Case 2 or Case 4), their temperature is higher, and when the patches are vegetation, their temperature is lower (Case 3). After sunrise, the solar radiation intensity gradually increases, and the surface picks up the sun's heat. As can be seen from the temperature-wind difference fields at 1000 LT and 1600 LT in Fig. 5, there are obvious patches of negative temperature difference in the corridor(Case 2–Case 1 and Case 4–Case 1). Although each group of patches in the corridors is similar in shape during the day, the patches are darker in the afternoon than in the morning. In contrast, there are no obvious temperature difference patches in the corridor of the group (Case 3–Case 1) during the daytime. That is, in both cases, temperatures were similar during the day. For both Case 2 and Case 4, the corridor surfaces warm their regions before sunrise and cool them after sunrise. The thermal properties of the corridor surface in Case 1 and Case 3 are very similar.

After sunset, due to the absorption of solar radiation during the day, the thermal effect of the land surface is more obvious, and the urban heat island effect is increasingly prominent. Since only case 1 in the 4 cases has no corridor, the wind speed of the other cases in part B (internal and external areas of the corridors) is higher than that of case 1:${\mathrm{Ws}}_{\rm{case }2/3}{-\mathrm{Ws}}_{\rm{case }1}\approx 2.0\mathrm{ m}/\mathrm{s}$, ${\mathrm{Ws}}_{\rm{case }4}-{\mathrm{Ws}}_{\rm{case }1}\approx 0.5\mathrm{ m}/\mathrm{s }$ (Fig. 5; 2000). In the case of higher wind speed, more heat will be blown downwind to some parts, so there are obvious negative temperature difference patches in part B of the different fields(Case 2–Case 1, Case 3–Case 1), and then positive temperature difference patches in part C (urban core area). In the southeast of the city, Case 1 is southeast wind, and Case 2 (Case 3 or Case 4) is south wind, which makes the heat of Case 2 (Case 3 or Case 4) in the suburb flow to part A, leading to positive temperature difference patches in part A. At 23:00, in the south of the temperature difference field, the temperature difference in and around the corridor is negative, that is, the temperature of Case 2, Case 3 and Case 4 is lower than that of Case 1. In the north of the temperature difference field gradually changes into a positive temperature difference. The more northward, the more obvious the positive temperature difference is, indicating that compared with case 1, the heat of Case 2, Case 3 and Case 4 is more blown to the north due to the wind (Fig. 5; 2300).

### B. Comparative analysis of daily average temperature curves in the corridor area and central urban area

In this study, the area within the second ring of Wuhan City is set as the central area of the city, and the area from corridor 1 to corridor 2 is set as the corridor area (see Fig. 4(1)). The temperature curve value is derived from the average value of each time point every day in the simulation period.

**Figure 6:**
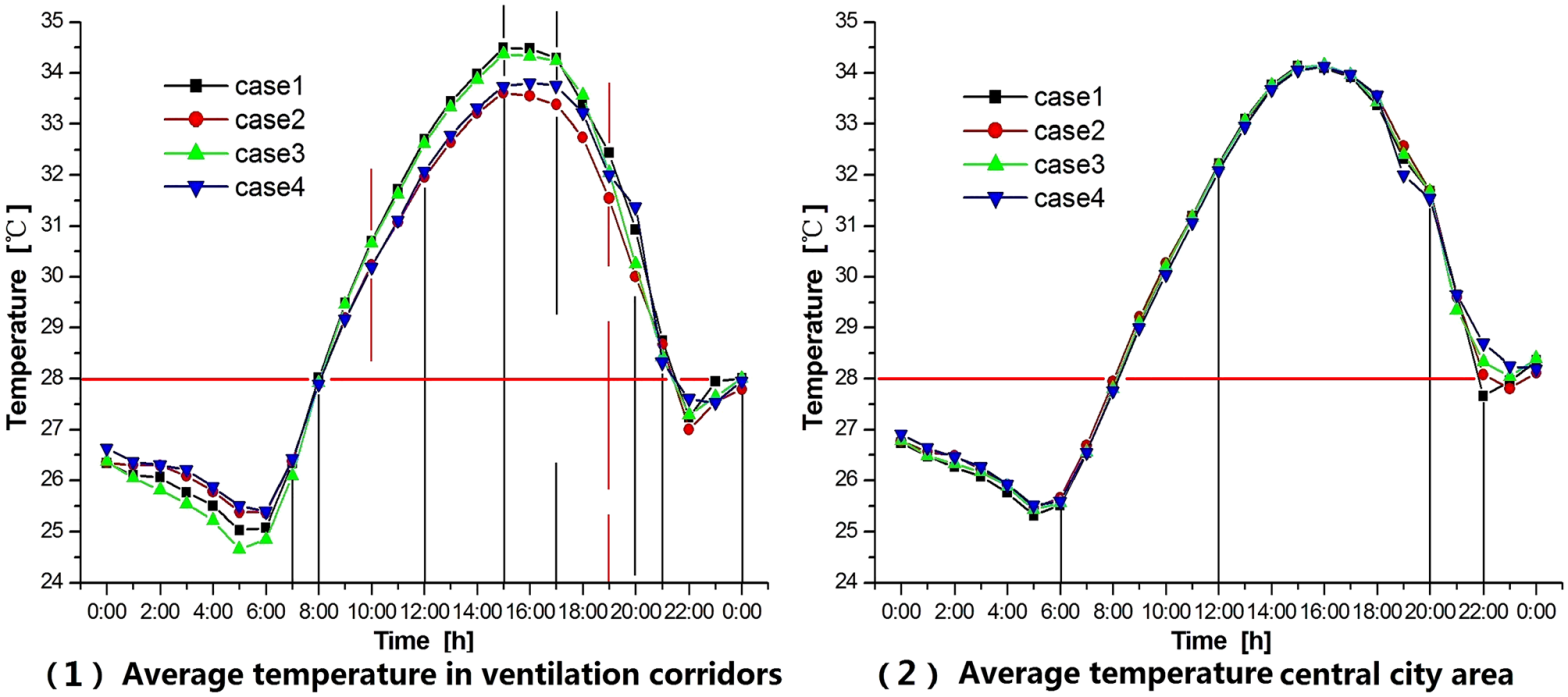
Average temperature curves of the corridor area and the central urban area.The image was generated by the research team using Origin 2021 (https://www.originlab.com). The data was extracted from the simulation results of the WRF–UCM model.

The average temperature curves of the corridor area are shown in Fig. 6(1). At 0700, 0800, and 2100 LT, the temperatures of all cases are approximately equal. From 0900 to 1500 LT, the most of time, the temperature curves show that ${\mathrm{T}}_{\rm{Case }1}\approx {\mathrm{T}}_{\rm{Case }3}>{\mathrm{T}}_{\rm{Case }2}\approx {\mathrm{T}}_{\rm{Case }4}$, and $0.8 \, {^\circ{\rm C} }\ge {\mathrm{T}}_{\rm{Case}\frac{1}{3}}-{\mathrm{T}}_{\rm{Case}\frac{2}{4}}\ge 0.3\, {^\circ{\rm C} }$. Especially at 1500 LT, the highest temperature of the day, and the temperature difference is obvious: ${\mathrm{T}}_{\rm{Case }1/3}-{\mathrm{T}}_{\rm{Case }2/4}\approx 0.8\, {^\circ{\rm C} }$. From 1600 to 1700 LT: ${\mathrm{T}}_{\rm{Case }1}\approx {\mathrm{T}}_{\rm{Case }3}>{\mathrm{T}}_{\rm{Case }4}>{\mathrm{T}}_{\rm{Case }2}$, ${\mathrm{T}}_{\rm{Case }1/3}-{\mathrm{T}}_{\rm{Case }2}\approx \, 0.8\, {^\circ{\rm C} }$, ${\mathrm{T}}_{\rm{Case }1/3}-{\mathrm{T}}_{\rm{Case }4}\approx 0.5 \, {^\circ{\rm C} }$. It can be seen that Case 2 and Case 4 show a good cooling effect on the corridor area during the sunshine period. From 1800 to 2000 LT ${\mathrm{T}}_{\rm{Case }2}$ keep the lowest temperature among the four cases. From 2100 to 2400 LT, the temperature curves of all cases are interwoven, and the temperature differences among the cases are not obvious. During the following 5 h (0100–0600 LT), the temperature differences among the cases are small, which is shown as: ${\mathrm{T}}_{\rm{Case }2}\approx {\mathrm{T}}_{\rm{Case }4}>{\mathrm{T}}_{\rm{Case }1}>{\mathrm{T}}_{\rm{Case }3}$. Among these four cases, the greening rate of the surface in the corridor of case 3 is the highest, and the heat release from the ground surface is the fastest, so the temperature is the lowest during this period, while in corridor Case 2 and Case 4, there are still water bodies, and the heat release are slower, so the temperatures are higher.

Figure 6(2) shows the average temperature curves of the central urban area. The temperature differences among the four cases are more obvious at 2200 LT than at other times of the day: ${\mathrm{T}}_{\rm{Case }4}>{\mathrm{T}}_{\rm{Case }3}>{\mathrm{T}}_{\rm{Case }2}>{\mathrm{T}}_{\rm{Case }1}$. From 0700 to 2000 LT, the temperature of Case 4 is slightly lower than that of other cases. From 0100 to 0500 LT, the temperatures of Case 1 and Case 3 are slightly lower than those of Case 2 and Case 4. The ventilation corridors of case 4 in the upwind position of the city can slightly improve the temperatures of the central urban area of Wuhan in the sweltering summer.

To summarize, Fig. 6 shows that Case 2 and Case 4 have good cooling effects in and around the corridors for more than 12 h per day. Case 4 can slightly improve the temperature in the central area of the city within 11 h of a day.

### C. Comparative analysis of the wind velocity curves

**Figure 7:**
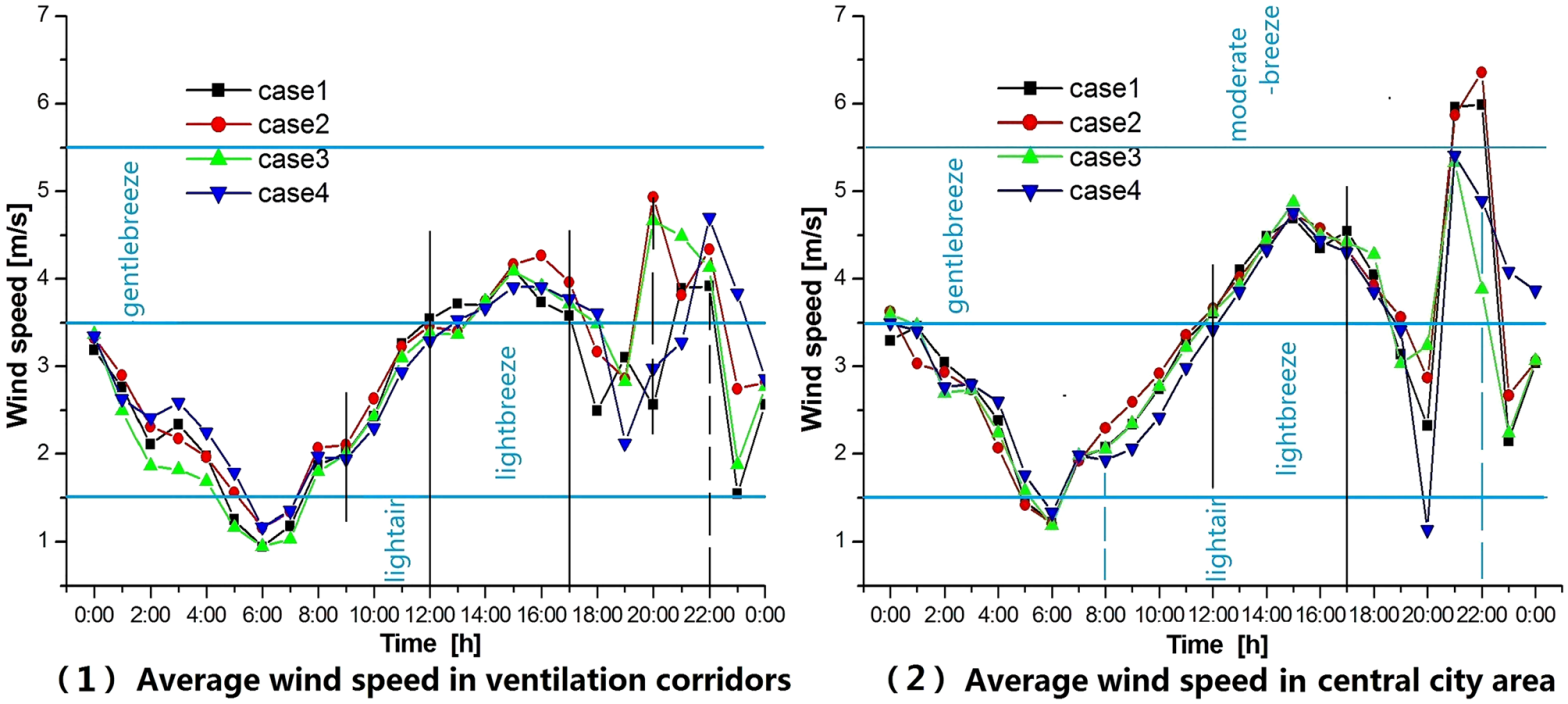
Average wind speed curves for the corridor and central city areas. The image was generated by the research team using Origin 2021 (https://www.originlab.com). The data was extracted from the simulation results of the WRF–UCM model.

Urban wind speed depends on seasonal climatic characteristics. However, the differences in wind speed among cases at the same time depend on the nature and form of the land cover. Figure 7 shows the average wind speed at each time in the simulation period, which shows that the all-day wind strength in Wuhan is generally weak in summer. From 1200 to 0000 LT, the average wind speed of all cases in the corridor area is lower than in the central city. From 0000 to 1700 LT in the corridor area and from 0000 to 1900 LT in the central urban area, the wind speed in the four cases is slightly different. From 1800 to 0000 LT, the land surface heat dissipation plays an important role in the urban microclimate, and the wind speed differences among cases are more significant. In the corridor area, the wind speed difference between Case 2 and Case 1 at 2000 LT is: ${\mathrm{Ws}}_{\rm{case }2}-{\mathrm{Ws}}_{\rm{case }1}\approx 2.5\mathrm{ m}/\mathrm{s}$; and the wind speed difference between Case 4 and Case 1 at 2300 LT is: ${\mathrm{Ws}}_{\rm{case }4}-{\mathrm{Ws}}_{\rm{case }1}\approx 2.5\mathrm{ m}/\mathrm{s}$. Compared with other cases, there are about 8 h (9000–2100 LT) with the weakest wind and about 10 h (1800–7000 LT) with the strongest wind in the corridor area of Case 4. It can be seen that the corridor form of Case 4 exerts the most significant influence on wind speed. The evening period, 1800–0000 LT, demonstrates an obvious urban wind system. During this time, the wind speed fluctuations are obvious for all cases in both the corridor and urban center areas, and the differences in wind speed among cases are also obvious.

In general, most of the day in summer, setting ventilation corridors only in the upwind part of the city has limited influence on the wind environment in the urban central area, and compared with the four cases, cases4 has a better effect.

### D. Comparison of average surface energy curves in the corridor area

**Figure 8:**
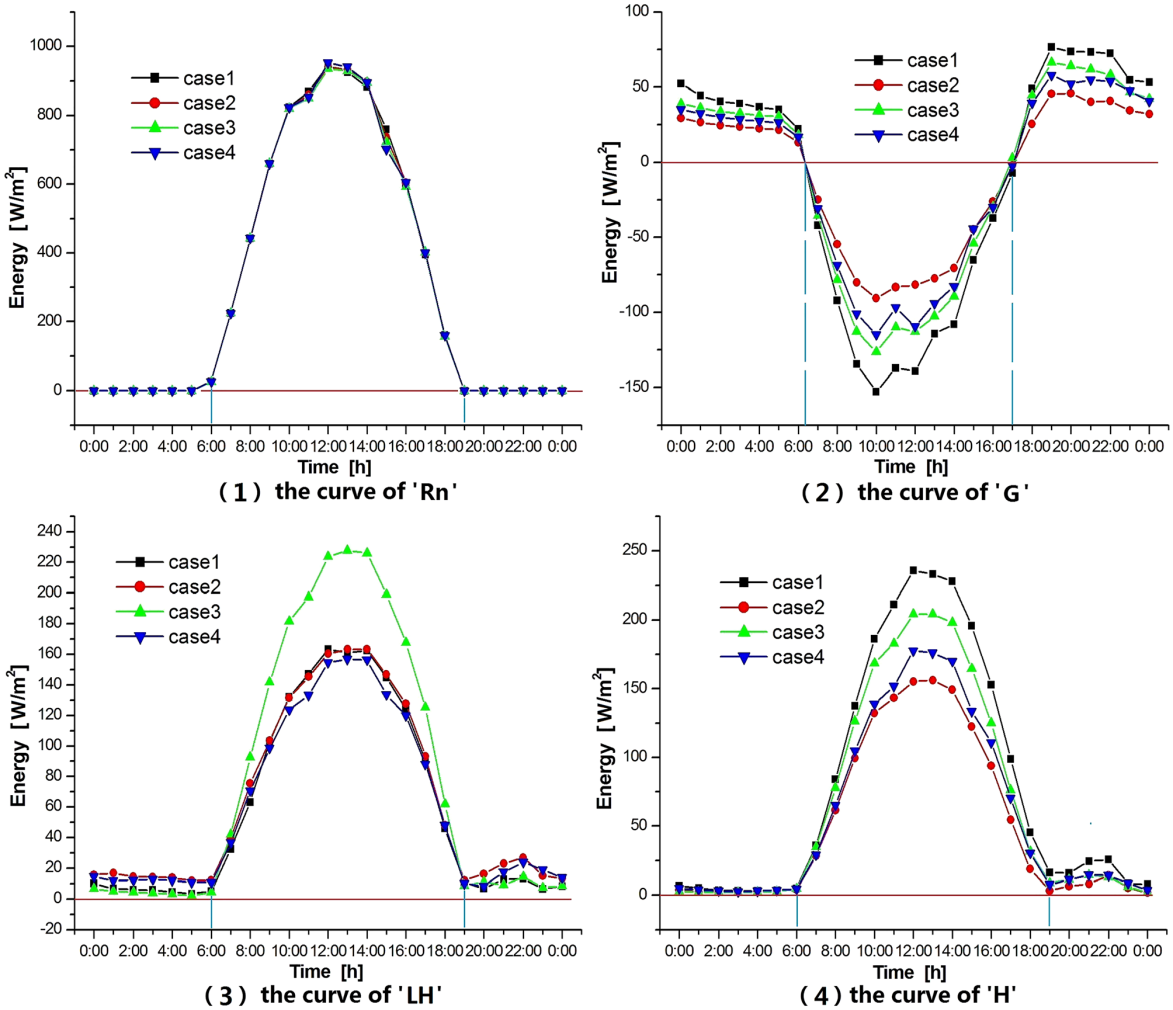
Comparison of energy curves.The image was generated by the research team using Origin 2021 (https://www.originlab.com). The data was extracted from the simulation results of the WRF-UCM model. *Rn* downward short wave flux at the ground surface, *G* ground heat flux, *LE* latent heat flux at the surface, *H* upward heat flux at the surface.

Figure 8 shows the average surface energy curves of the four cases in the corridor area. The abbreviations used in this figure are clarified below.

Rn: downward short wave flux at the ground surface. It can be seen from Fig. 8(1) that the Rn values of all cases are basically the same.

G: ground heat flux(the change of heat storage inside the underlying surface). G varies from case to case. From 0600 to 1700 LT, the ground is in the state of absorbing heat; from 1700 to 0600 LT, the ground is in the state of releasing heat. Recall that the land surfaces in the corridors of Case 2 are mainly water, which has larger thermal inertia and specific heat capacity, slower heat exchange, and the least gain and loss of energy per unit of time. It follows that ${\mathrm{G}}_{\rm{case }2}$ is the smallest among the four cases. The surface of the corridors of Case 1 is construction land, which exchanges heat from three dimensions, and has the most energy gain and loss, so ${\mathrm{G}}_{\rm{case }1}$ is the largest. The G value of the four cases can be ranked as follows:$\left|{\mathrm{G}}_{\rm{case }1}\right|>\left|{\mathrm{G}}_{\rm{case }3}\right|>\left|{\mathrm{G}}_{\rm{case }4}\right|>\left|{\mathrm{G}}_{\rm{case }2}\right|$ (Fig. 8(2)).

LH: latent heat flux at the surface(Heat exchange per unit area under constant temperature). The corridors of Case 3 consist of low-density construction land and abundant vegetation. During the daytime in summer, the LH value of Case 3 was significantly higher than that of other cases due to transpiration and evapotranspiration under strong sunshine conditions (Fig. 8(3)). In summer, the air humidity in Wuhan is high, and the humidity of the water surface is higher than that of other surfaces, which directly affects the water evapotranspiration. It also affects the heat flux from the water vapor phase transition to the atmosphere. Therefore, the LH values of the two cases (Case 2 and Case 4) with water bodies in the corridors are not higher than those of the other cases.

H: upward heat flux at the surface (the sensible turbulent heat flux), the value of which can reflect the increase or decrease of air temperature. From 0000 to 0600 LT the H values of the four cases are approximately the same. There is a slight difference in H values among the four cases from 1900 to 2400 LT. In other periods, the differences of H value among the four cases obviously changed from weak to strong, from strong to weak. The H values from 0600 to 2400 LT can be ranked as:${\mathrm{H}}_{\rm{Case }1}>{\mathrm{H}}_{\rm{Case }3}>{\mathrm{H}}_{\rm{Case }4}>{\mathrm{H}}_{\rm{Case }2}$, which is basically consistent with the order of temperature values in the same period (Figs. 8(4) and 6(1)). Temperature T2 is an intuitive reflection of H, and the greater the value of G, the greater the impact on T2, although this phenomenon is delayed.

In summary, the surfaces within the corridors in all four cases receive the same short-wave radiant energy. Throughout the day, Case 1 has the highest Ground heat flux value (${\mathrm{G}}_{\rm{Case }1}$) compared to the other cases. The latent heat flux of Case 3 (${\mathrm{LH}}_{\rm{Case }3}$) is obviously the largest during the day, while that of Case 3 (${\mathrm{LH}}_{\rm{Case }3}$) is slightly smaller than others at night. The LH values of the four cases are similar. The sensible heat flux of Case 1(${\mathrm{H}}_{\rm{Case }3}$) is obviously the largest in the daytime, and that of Case 1(${\mathrm{H}}_{\rm{Case }1}$) is slightly larger than other values in the night. The H values of the four cases are similar too.

The land surface heat balance equation is: ${\mathrm{R}}_{\rm{n}}+{\mathrm{A}}_{\rm{n}}=\mathrm{G}+\mathrm{H}+\mathrm{LH}+{\mathrm{Q}}_{\rm{A}}$. ${\mathrm{A}}_{\rm{n}}$: anthropogenic heat (it will become larger as the intensity of construction land increases); ${\mathrm{Q}}_{\rm{A}}$: thermal advection (since the advection heat entering the urban canopy is equal to the advection heat expended, so ${\mathrm{Q}}_{\rm{A}}$ can be ignored). In the corridors of the four cases, the same shortwave radiation is obtained in the same period, the ${\mathrm{R}}_{\rm{n}}$ values in the corridor regions of the four cases are equal. The sensible heat flux $\mathrm{H}$ value, which reflects the temperature condition, is determined by the three variables on both sides of the equation: ${\mathrm{A}}_{\rm{n}}$, $\mathrm{G}$, and $\mathrm{LH}$. The values of these three variables are determined and affected by many factors such as climatic conditions, land nature and use intensity. Take the two plots of the same area as an example, when their surface features are the same, their ${\mathrm{R}}_{\rm{n}}$ and $\mathrm{G}$ values are equal. If we assume that their ${\mathrm{A}}_{\rm{n}}$ values are the same, then the $\mathrm{H}$ value is negatively correlated with the $\mathrm{LH}$ value, and if their $\mathrm{LH}$ values are the same, then the $\mathrm{H}$ value is positively correlated with the ${\mathrm{A}}_{\rm{n}}$ value. However, the values of $\mathrm{G}$, $\mathrm{H}$, $\mathrm{LH}$ and ${\mathrm{A}}_{\rm{n}}$ in the four corridors are different respectively, and the four values($\mathrm{G}$, $\mathrm{H}$, $\mathrm{LH}$ and ${\mathrm{A}}_{\rm{n}}$) in each corridor influence and restrict each other, with complex physical relationships, which leads to the differences in microclimate among the four cases. WRF + UCM model coupled with multi-physics scheme solves this problem well.

### Discussion

Based on the WRF-UCM model, this paper explores the thermal environment regulation ability of different corridor modes by setting up four simulation cases in southeast Wuhan. The results show that different corridor modes have different thermal regulation capabilities. The corridor mode with more water bodies, green spaces and other natural underlying surfaces is better than that with more artificial underlying surfaces. This phenomenon is related to the different properties of the underlying surface. Different underlying surface properties will lead to different energy fields and wind fields in the corridor area, resulting in different urban temperature fields.

First, in terms of the impact on the energy field. The thermal inertia and specific heat capacity of water are greater than those of natural surfaces^31^ such as forest land and grassland, and approximately equal to those of artificial surfaces such as cement, mortar, and asphalt. However, the water body has obvious transpiration, and the water body can only exchange plane (surface) heat, and the construction land is three-dimensional heat exchange. Therefore, the amount of heat radiation absorbed and lost by the water body is smaller than that absorbed and lost by the construction land. Due to the strong transpiration of vegetation during the day, the corridor model with green space will have a lower heat radiation exchange capacity than the underlying surface of the water body, but higher than the underlying surface of the construction. Therefore, compared with the corridor mode with only construction land, the corridor mode with water and green space has a lower temperature during the day and a stronger ability to regulate the thermal environment.

Secondly, in terms of the impact on the wind field. Compared with natural underlying surfaces such as water and vegetation, artificial underlying surfaces such as construction land have higher roughness and average height. This will block the external wind and intensify the weakening of air flow. As a result, the heat in the city cannot be dissipated quickly. The corridor model with more water and vegetation has a higher wind speed, so it can dissipate heat faster.

In terms of exploring the thermal environment regulation ability of different corridor modes, there are few research results directly related to this paper in the academic community. However, many studies have shown that water, green space and other blue-green spaces have the function and way to alleviate the local thermal environment of the city. And the area of blue-green space has a positive correlation with this thermal environment regulation ability^32,33^. It can be seen that reserving a certain area of water and green space during the construction of ventilation corridors does indeed improve the thermal environmental regulation capacity of the corridors. At present, there is no view in the academic circle that increasing the artificial underground surface such as construction land will bring optimization effect to the local thermal environment. On the contrary, scientists believe that the increase of building land often leads to the deterioration of the urban thermal environment^34,35^. Based on the research results of this paper, the follow-up work can further explore the optimal ratio of water, green space and construction land in the construction of the optimal corridor mode.

The research results of this paper can provide reference and guidance for scholars' related research on ventilation corridor, but there are also some limitations. First, in this paper, only four simulation cases of corridor modes are set up, and the types of corridor modes are fewer. In the follow-up study, the simulation cases of the experimental group with multiple corridor modes can be added for optimization. The second is the simulation accuracy of WRF–UCM model. UCM model only divides urban land into three land use intensities, which is difficult to accurately express the spatial distribution differences of urban thermal environment. In the future, the simulation accuracy of the model can be improved by increasing the number of urban land types in the UCM model.

## Conclusions

Through the analysis of temperature-wind difference fields, average temperature curves, average wind speed curves, and energy curves, the following conclusions can be obtained:

1. Due to the different physical responses to solar radiation, the surface properties and surface morphology in ventilation corridors affect the microclimate of the city.
2. In a whole day, the temperature of Case 2 and Case 4 in the corridor area is basically the same for most of the time. Compared with other cases, the temperature of the two cases is lower in the corridor area during the period of higher temperature in the day (from 9000 to 1900 LT), and the maximum difference reached 0.8 ℃ at 15:00, showing that the two corridores with water bodies have better cooling effects. At the same time, the corridor of Case 4 can slightly improve the temperature condition in the city center.
3. From midnight to early morning, Case 3 with lower construction land intensity and higher vegetation coverage in the corridor dissipates heat faster than Case 1, resulting in lower temperature of Case 3 there. It can be seen that transpiration of green space can regulate the nighttime microclimate environment in summer. Ecological patch arrangement is also an effective way to improve urban microclimate environment.
4. The wind speed curves of the corridor area and the urban center area show that different surface friction forces originate from different underlying surface forms. Among the four cases, Case 4 has the most obvious impact on wind speed. Especially during the period when the wind becomes weaker (from 22:00 to 7:00), the wind speed in Case 4 is much stronger than in other cases.
5. Each element in the surface heat balance equation has a complex physical relationship of mutual influence and restriction. WRF + UCM calculation shows that ${\mathrm{G}}_{\rm{Case }1}$ is the largest a whole day, $\mathrm{LH}$ and $\mathrm{H}$ are the largest in the day, $\mathrm{LH}$ and $\mathrm{H}$ are similar in the four cases from night to morning, in all four cases, the ordering of T2 values is positively correlated with the ordering of G and H values.

The above analysis shows that the corridors of Case 2 and Case 4 can better regulate the temperature of the corridor region in Wuhan in summer. The corridors of Case 4 are slightly improved on the thermal environment of the urban center and are most sensitive to the effects of wind speed. Especially during the period when the wind becomes weaker (from night to morning), the wind speed in Case 4 is much stronger than in other cases. As the largest transportation hub city in inland China, Wuhan has a very high economic value of land, so it is important to take into account both climate regulation and urban land utilization. Compared with Case 2, Case 4 not only improves the urban thermal environment in summer, but also makes use of part of the land in the corridor. Therefore, we believe that Case 4 is the first choice of the four corridor forms in the southeast of Wuhan.

## Data Availability

In this study, the data of numerical model simulations are too large to archive or to transfer. Therefore, we provide all the information needed to replicate the simulations. WRF model version 3.6 was adopted in this study. The model code, compilation script, initial and boundary condition files, and the namelist settings are available at https://download.csdn.net/download/baidu_41819002/79220552.
